# Out of the blue: blue light mediates ascorbate synthesis

**DOI:** 10.1093/plcell/koad109

**Published:** 2023-04-12

**Authors:** Maryam Rahmati Ishka

**Affiliations:** Assistant Features Editor, The Plant Cell, American Society of Plant Biologists; Boyce Thompson Institute, Ithaca, NY, USA

Ascorbate (vitamin C) is an antioxidant found in fresh fruits and vegetables that is an essential nutrient in the human diet. The content of ascorbate fluctuates with environmental changes and can influence reactive oxygen species production. The high antioxidant capacity of ascorbate makes it a good target for breeding nutrition-dense fruits. Although the L-galactose pathway of ascorbate synthesis in plants is well established, little is known about how the pathway is regulated. In this issue, **Céline Bournonville, Kentaro Mori, and colleagues (Bournonville et al. 2023)** found that ascorbate synthesis in tomato (*Solanum lycopersicum*) is regulated by a flavin-dependent photoreceptor named PAS/LOV Protein (PLP), which inhibits a key step in the pathway except when blue light is present (see Fig.).

**Figure.**
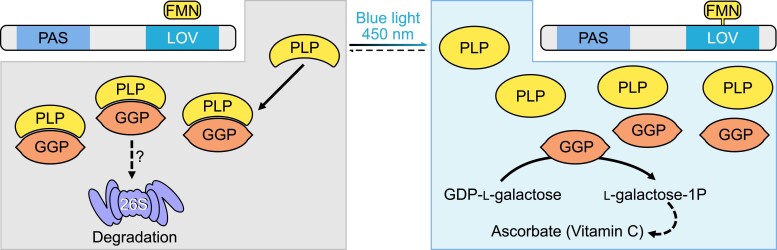
Blue light–mediated regulation of the PLP-GGP interaction regulates ascorbate synthesis. PLP protein binds GGP, leading to its deactivation. However, blue light exposure deactivates PLP, leading to activation of GGP and thus ascorbate accumulation. Adapted from Bournonville et al. (2023), Figure 7.

Light is a critical environmental factor that stimulates ascorbate accumulation by affecting gene expression of GDP-L-galactose phosphorylase (GGP), a key enzyme of the ascorbate biosynthesis pathway (Fenech et al. 2021). GGP-related mutants in Arabidopsis (*Arabidopsis thaliana*) display a dramatic decrease in ascorbate content and are unable to grow without exogenous application of ascorbate (Dowdle et al. 2007). In addition to its transcriptional regulation by light, GGP activity also appears to be regulated at the protein level. However, how GGP proteins and light are involved in the direct regulation of ascorbate synthesis is not known.

Using a previously identified EMS mutant with increased ascorbate content (Deslous et al. 2021), the authors mapped a mutation associated with the increased ascorbate within the *PLP* gene. By generating a *plp* mutant in wild-type tomato using CRISPR/Cas9, the authors established that the mutant shows high ascorbate content in all organs compared with wild-type plants, and PLP genetically functions as a repressor of ascorbate accumulation in tomato throughout multiple developmental stages. To address the mechanism of this regulation, the authors tested the possibility that PLP and GGP proteins interact first by examining their subcellular localization using transient expression in tobacco (*Nicotiana - benthamiana*) leaves. Confocal microscopy images showed that both proteins possess a dual residency pattern that marks both the nucleus and cytosol. Secondly, using the Bimolecular Fluorescence Complementation assay, they found that indeed PLP interacts with GGP in both the nucleus and cytosol. Moreover, this interaction was abolished using a truncated version of the PLP protein that lacks the blue light–sensing domain, suggesting that the PLP-GGP protein-protein interaction might be blue light dependent.

To further investigate how the blue light–sensing LOV domain in PLP participates in this PLP-GGP interaction, the authors used a heterologous system in mammalian cells. Unlike the truncated PLP, a strong interaction was detected between full-length PLP and GGP in the dark, whereas the blue light exposure minimized the interaction. The use of this heterologous system suggested that no additional factor is required for the PLP-GGP interaction, providing evidence for the blue light–mediated interaction of the PLP and GGP. Further, using the activity assay in vitro, the authors showed that the blue light–mediated interaction inhibits GGP enzyme activity.

Together, Bournonville et al. (2023) provide mechanistic insights into the role of blue light as a direct factor in ascorbate synthesis by suppression of the photoreceptor PLP, identified as a negative regulator in ascorbate synthesis. Suppression of PLP leads to activation of GGP and ascorbate accumulation, which, in turn, mitigates blue light–mediated oxidative damage. In the future, it would be intriguing to investigate the stoichiometry of the 2 proteins using crystallography.
